# A machine learning interpretation of the contribution of foliar fungicides to soybean yield in the north‐central United States

**DOI:** 10.1038/s41598-021-98230-2

**Published:** 2021-09-21

**Authors:** Denis A. Shah, Thomas R. Butts, Spyridon Mourtzinis, Juan I. Rattalino Edreira, Patricio Grassini, Shawn P. Conley, Paul D. Esker

**Affiliations:** 1 Department of Plant Pathology, Kansas State University, Manhattan, KS 66506 USA; 2 Department of Crop, Soil, and Environmental Sciences, University of Arkansas System Division of Agriculture, Lonoke, AR 72086 USA; 3 Agstat Consulting, Athens, Greece; 4 Department of Agronomy and Horticulture, University of Nebraska-Lincoln, Lincoln, NE 68583 USA; 5 Department of Agronomy, University of Wisconsin-Madison, Madison, WI 53706 USA; 6 Department of Plant Pathology and Environmental Microbiology, Pennsylvania State University, University Park, PA 16802 USA

**Keywords:** Agroecology, Environmental sciences

## Abstract

Foliar fungicide usage in soybeans in the north-central United States increased steadily over the past two decades. An agronomically-interpretable machine learning framework was used to understand the importance of foliar fungicides relative to other factors associated with realized soybean yields, as reported by growers surveyed from 2014 to 2016. A database of 2738 spatially referenced fields (of which 30% had been sprayed with foliar fungicides) was fit to a random forest model explaining soybean yield. Latitude (a proxy for unmeasured agronomic factors) and sowing date were the two most important factors associated with yield. Foliar fungicides ranked 7th out of 20 factors in terms of relative importance. Pairwise interactions between latitude, sowing date and foliar fungicide use indicated more yield benefit to using foliar fungicides in late-planted fields and in lower latitudes. There was a greater yield response to foliar fungicides in higher-yield environments, but less than a 100 kg/ha yield penalty for not using foliar fungicides in such environments. Except in a few production environments, yield gains due to foliar fungicides sufficiently offset the associated costs of the intervention when soybean prices are near-to-above average but do not negate the importance of disease scouting and fungicide resistance management.

## Introduction

Soybean (*Glycine max*) is one of the major crops produced in the United States (U.S.), planted on an estimated 33.9 million ha in 2020^1^. Success in growing soybean depends on multiple management decisions, which rest largely on the individual grower or crop manager, including choice of cultivar^2,3^, sowing date^4,5^, row width and seeding rate^6^, seed treatments^7–9^, herbicide program^10^, nutrient fertilization^11,12^, irrigation^13^, drainage^14^, crop rotation and tillage^15,16^, and foliar fungicide and/or insecticide application^17–21^.

The decade from 2005 to 2015 saw the use of foliar fungicides in U.S. soybeans double on a per unit area basis (g of product applied per ha), and almost triple in terms of total product applied (tonnes) across all so-treated fields^22^. Foliar fungicide applications are not necessarily made in response to the actual threat or presence of diseases; prophylactic applications may be made to the perceived future possibility of disease (sometimes as an insurance spray) or for so-called plant health benefits (e.g., a “greening effect”^23^). The accumulated body of evidence to date does show that foliar diseases are responsible for measurable financial losses^24^. Yet at the same time, foliar diseases in soybean are, except in a few circumstances, rarely severe when compared to losses due to soilborne pathogens^25,26^. When foliar diseases are absent or at low levels, the consensus from recent field trials is that the yield response to foliar fungicides (including the plant health benefit effect) are not sufficient to offset the interventional costs^16,17,19–21,27–30^.

The increase in foliar fungicide use in U.S. soybeans does therefore seem to contradict the scientific research showing low economic returns when disease levels are low or absent. A partial explanation may be that research moves slower than the adoption of a practice by growers responding to changing economic or marketing forces^31^. The myriad of soybean crop management choices makes it impossible to account for complexity beyond three-way interactions in designed field trials^30,32^ which are by practical necessity focused on a few controlled main effects of interest. Moreover, such trials are conducted in a few locations at best, which raises questions about the scalability of inference beyond local conditions. Therefore, it is not uncommon for inferences made from research trials to conflict across studies, and these inferential discrepancies are often a point of discourse in many agronomically-based papers. For example, in three different sets of field experiments in the U.S., foliar fungicides increased soybean yield in only three out of 11 site-years^33^, four out of 12 site-years^20^, and one out of 16 site-years in the investigated production systems^29^. In other studies, there was little to no significant effect on soybean yield from foliar-applied fungicides^17,34,35^.

A novel complementary approach to traditional field experiments, given their limited design and inferential space, uses grower-supplied data linked in a spatial framework to other data layers representing soil properties and weather. The format is expandable as more layers or data become available^36^. This approach leads to an observational database covering wide and diverse geographies, is broad in scope, and possibly capturing complex, realistic interactions among agronomic, environmental and crop management variables beyond those which may be represented in designed field trials. The challenge, however, is that the multidimensional observational space must now be queried for pattern recognition and for drawing inferences from those identified relationships. This usually requires a machine-learning (ML) approach rather than traditional statistical methods^37^.

Traditional statistical models are associated with being interpretable, which in the present context means being able to understand, from the human perspective, how each predictor contributes to soybean yield (or loss); whereas ML algorithms can be criticized as being opaque (i.e., “black box”^38^). However, recent advances in ML interpretation^39^ are removing the black box label, so that this class of models, usually associated with predictive performance, is becoming more explainable as well. Trust in a model (i.e., understanding *why* a prediction was made) is a very important criterion to stakeholders^39^. In this paper, a ML algorithm was used to fit a yield prediction model to a grower-derived database on soybean production practices in the north‐central U.S. The model was then queried with the objective of understanding how foliar fungicides fit into overall soybean production practices in the north-central U.S. and their contribution to yield from an economic standpoint.

## Results and discussion

The surveyed, rainfed commercial soybean fields were spread across the U.S. north-central region (Supplementary Fig. S1 online) with a latitudinal gradient evident for maturity group (MG). The number of fields (*n*) was distributed evenly across the three years (2014: *n* = 812, 2015: *n* = 960, 2016: *n* = 966). Among the 2738 fields, 833 (or 30.4%) were sprayed with foliar fungicides. Out of the 833 fields sprayed with foliar fungicides, 623 (74.8%) had also been sprayed with foliar insecticides.

A *t*-test estimate of the yield difference between all fields sprayed with foliar fungicides and those which were not was 0.46 t/ha (95% confidence interval [CI] of 0.39 to 0.52 t/ha). When *t*-tests were applied to fields within TEDs (the 12 TEDs with the most fields), half of the 95% CIs included zero, indicative of possibly no yield increase due to foliar fungicides over unsprayed fields in those TEDs (Supplementary Fig. S2 online). A linear mixed model with random slopes and intercepts for the fungicide effect within TEDs returned an estimated yield gain of 0.33 t/ha due to foliar fungicide use. A simpler model without random slopes for foliar fungicide was a worse fit to the data. Together these basic tests were indicative of heterogenous effects concerning foliar fungicides and yield gain, implying other global (regional) and local (field specific) conditions may be involved as factors.

A tuned random forest (RF) model fitted to the entire dataset (all 2738 observations) overpredicted soybean yield at low actual yields, and underpredicted at the high-yield end (Supplementary Fig. S3 online). However, as 99% of the residual values were less than or equal to |0.25 t/ha| which corresponded to less than 7% of the average yield, we proceeded with the interpretation of the fit RF model. The mean predicted soybean yield (global average) was 3.79 t/ha (minimum = 1.13 t/ha, maximum = 6.02 t/ha, standard deviation = 0.81 t/ha, root mean squared error between the observed and predicted yields = 0.1 t/ha).

**Figure 1:**
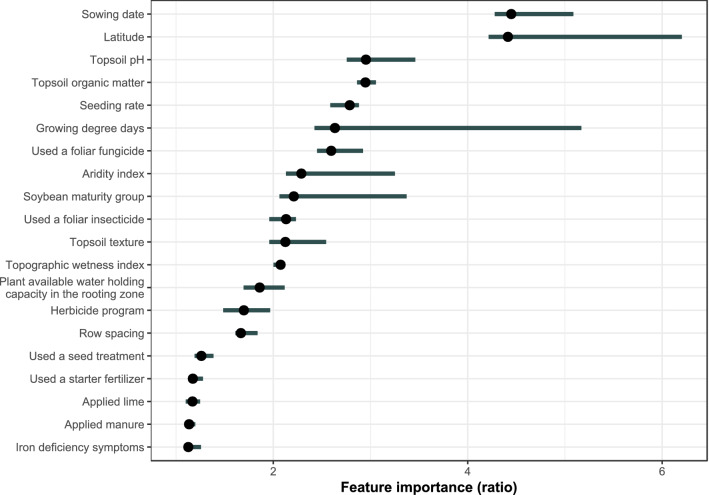
Importance of management-based variables in a random forest model predicting soybean yield. Feature importance was measured as the ratio of model error, after permuting the values of a feature, to the original model error. A predictor was unimportant if the ratio was 1. Points are the medians of the ratio over all the permutations (repeated 20 times). The bars represent the range between the 5% and 95% quantiles. Sowing date was the number of days from Jan 01. Growing degree days and the aridity index were annualized categorical constructs used within the definition of technology extrapolation domains (TEDs). Foliar fungicide or insecticide use, seed treatment use, starter fertilizer use, lime and manure applications were all binary variables for the use (or not) of the practice. Iron deficiency was likewise binary (symptoms were observed or not). Topsoil texture, plant available water holding capacity in the rooting zone, row spacing, and herbicide program were categorical variables with five, seven, five, and four levels, respectively.

At the global model level, location (latitude; a surrogate for other unmeasured variables) and sowing date (day of year from Jan 01) were the two variables most associated with yield (Fig. 1), consistent with the central importance of early planting to soybean yield^5,13^. Soil-related properties (pH and organic matter content of the topsoil) were also associated with yield (Fig. 1). Management-related variables such as foliar fungicide, insecticide and herbicide applications were of intermediate importance, and other management variables (row spacing, seed treatments, starter fertilizer) were on the lower end of the importance spectrum in predicting soybean yield (Fig. 1). Insecticide and fungicide seed treatments were poorly associated with soybean yield increases as has been previously shown^8,40^. The relatively lower importance of row spacing is consistent with previous analyses of this variable from soybean grower data^6^. The dataset we analyzed did not contain enough observations to include artificial drainage as a variable, which has been shown to influence soybean yield, presumably by allowing earlier sowing^14^.

**Figure 2:**
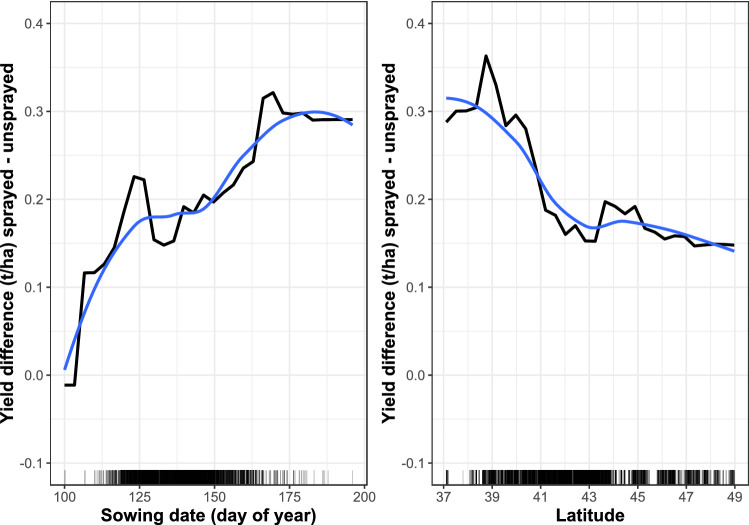
Two-way partial dependence plots of the global effects of (i) foliar fungicide use and sowing date (left panel), and (ii) foliar fungicide use and latitude (right panel) on soybean yield. The black plotted curves are the yield differences between fields that were sprayed or not sprayed with foliar fungicides. Smoothed versions of the curves are shown in blue.

The strongest pairwise interactions included that between sowing date and latitude. Delayed sowing at higher latitudes decreased yield by about 1 t/ha relative to the highest yielding fields sown early in the more southerly locations (Supplementary Fig. S4 online). Further examination of the interactions showed that the yield difference between sprayed and unsprayed fields increased with later sowing, indicative of a greater fungicide benefit in later-planted fields (Fig. 2). This would seem to conflict with the results of a recent meta-analysis in which soybean yields responded better when foliar fungicides were applied to early-planted fields^27^, but in that study there was also the confounding effect of higher-than-average rainfall between sowing and the R3 growth stage. With respect to latitude, the global difference in yield between sprayed and unsprayed fields decreased as one moved further north (Fig. 2), suggesting that foliar fungicides were of more benefit when applied to the more southerly located fields, which do tend to experience more or prolonged conditions conducive to foliar diseases than the northern fields^22,24^.

**Figure 3:**
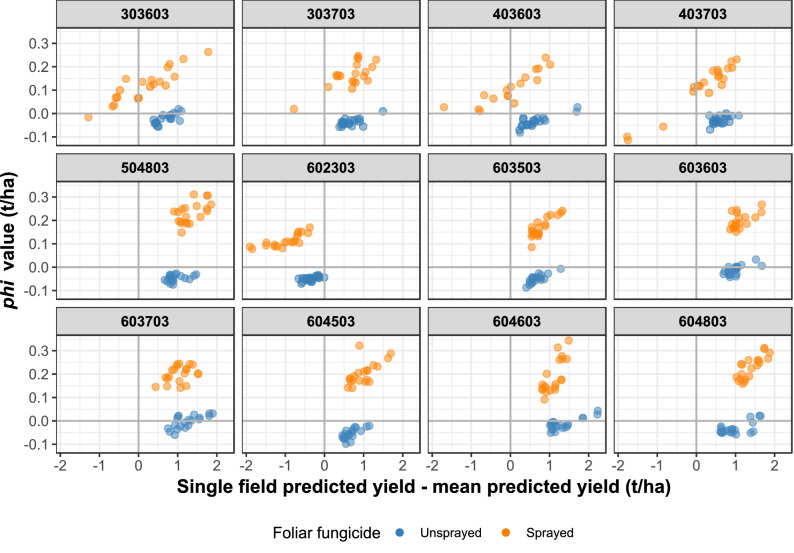
Shapley *phi* values attributed to foliar fungicide use for two cohorts of fields within the 12 technology extrapolation domains (TEDs) with the most fields. Within each TED, the cohorts are the 20 highest-yielding fields among those sprayed with foliar fungicides and the 20 highest-yielding fields among those which were unsprayed.

Focusing on model interpretation at the local level, we examined the Shapley φ values (see the “Methods” section for more information) associated with foliar fungicide applications for different subsets (*s*) and cohorts (*c*) of fields within the data (see Supplementary Table S1 online). The 1st subset (*s*_1_) was comprised of the 20 highest-yielding fields among those sprayed with foliar fungicides (*s*_1_*c*_1_) and the 20 highest-yielding fields among those which were not sprayed (*s*_1_*c*_2_) in each of the 12 technology extrapolation domains (TEDs) in the data matrix with adequate numbers of fields for comparisons (see also Supplementary Table S2 online; Supplementary Fig. S5 online maps the field locations within these 12 TEDs). A TED is a region (not necessarily spatially contiguous) with similar biophysical properties^41^. Predicted yields within these cohorts were mainly above the global average of 3.79 t/ha, except in TED 602303 (Fig. 3), which corresponded to fields in North Dakota (Supplementary Fig. S5). In most cases Shapley φ values for foliar fungicide use exhibited a positive contribution to the yield above the global average. If these cohorts of fields represented high-yielding environments within each TED, then foliar fungicide sprays contributed positively up to 0.3 t/ha in the yield increase above the global average in *s*_1_*c*_1_. However, among high-yielding fields in *s*_1_*c*_2_, the penalty for *not* spraying was less than 0.1 t/ha. This finding supports the contention that fungicide sprays are most worthwhile in high-yielding environments. Supplementary Fig. S6 online complements Fig. 3 by summarizing the Shapley φ values in another visual format. The overall mean predicted yield for the unsprayed (*s*_1_*c*_2_) fields was slightly higher (by 0.1 t/ha) than that for the sprayed (*s*_1_*c*_1_) fields (Supplementary Fig. S6 online). This difference may have been driven by the higher variability in yields among the two cohorts (particularly for TEDs 403603, 602303, 403703, and 303603), or underlying differences in other management factors. Also, the number of sprayed fields in each of these four TEDs was at the target sampling boundary of 20 fields per TED (Supplementary Table S2 online).

**Figure 4:**
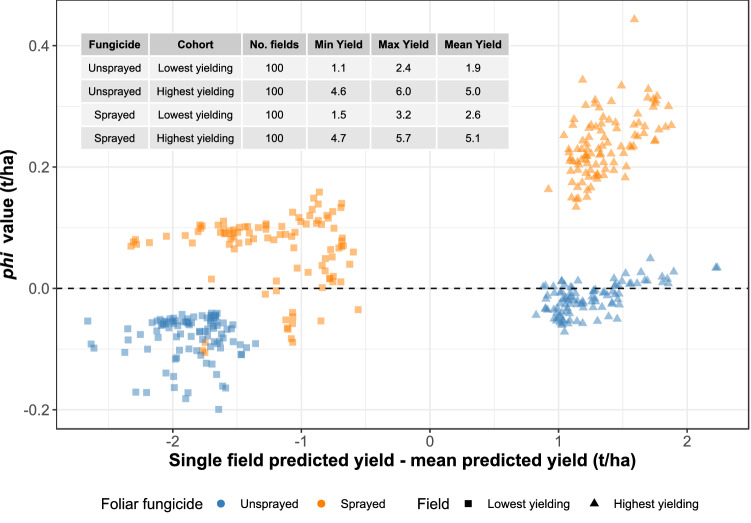
Shapley *phi* values attributed to foliar fungicides for four cohorts of soybean fields. The cohorts are (i) the 100 highest-yielding fungicide-treated fields, (ii) the 100 lowest-yielding fungicide-treated fields, (iii) the 100 highest-yielding unsprayed fields, and (iv) the 100 lowest-yielding unsprayed fields. The insert table summarizes the minimum (Min), maximum (Max) and mean predicted yields (t/ha) for each of the four cohorts. Point color represents whether fields were sprayed or unsprayed, whereas point shape represents whether fields were in the lowest-yielding or highest-yielding cohorts.

The Shapley φ values for fungicide use were well-separated among the four cohorts of fields of *s*_2_ (Fig. 4, Supplementary Table S1 online). The fields within *s*_2_ were selected across the entire dataset and not by TED membership. The lowest-yielding fields (*s*_2_*c*_2_ & *s*_2_*c*_4_) were all below the global yield average, whereas the converse was true of the highest-yielding fields (*s*_2_*c*_1_ & *s*_2_*c*_3_). Among the lowest-yielding fields, foliar fungicides were mainly associated with a positive, but less than 0.2 t/ha, effect on yield (*s*_2_*c*_2_), and other factors were responsible for dropping a field’s yield to below the global average. Amongst the highest-yielding fields (*s*_2_*c*_1_), foliar fungicides were associated with between 0.15 and 0.35 t/ha of the yield above the global average. These Shapley φ values for the contribution of foliar fungicides are consistent with estimates of the yield response to foliar fungicides from a meta-analytic perspective^27^. Given that the individual yields in *s*_2_*c*_1_ & *s*_2_*c*_3_ were 1 to 2 t/ha above the global average, other location-driven factors such as early sowing (Fig. 1) were the larger drivers of yield in these cases. However, there was only a negligible or small (< 0.1 t/ha) penalty for not using foliar fungicides in high-yield situations (*s*_2_*c*_3_; see also Supplementary Fig. S7 online).

**Figure 5:**
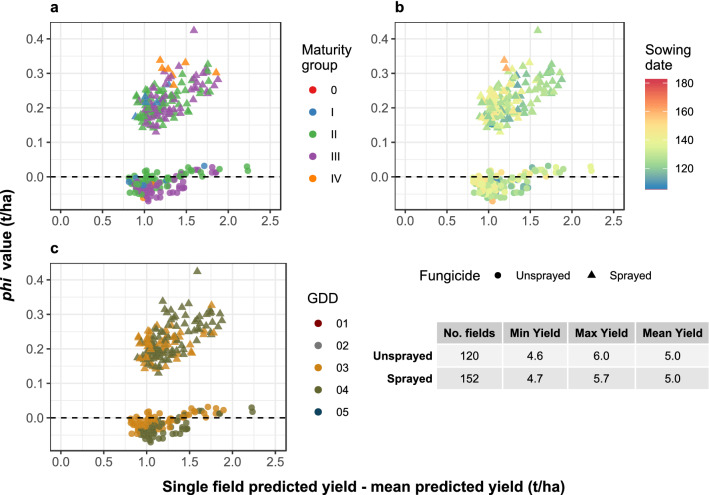
Shapley *phi* values attributed to foliar fungicides for two cohorts of high-yielding soybean fields: the 90th percentile for yield among fungicide-treated (sprayed) fields, and the 90th percentile for yield among unsprayed fields. Point shape indicates whether the field was treated with fungicides (Sprayed) or not (Unsprayed). Data points are colored by (**a**) soybean maturity group, (**b**) sowing date, as the number of days from Jan 01, (**c**) growing degree days (GDD), as defined in the TED construct. Five GDD categories were represented in the data, although only two of those (03, 04) were present in the cohorts plotted. 01 = 0 to 2670 °C; 02 = 2671 to 3169 °C; 03 = 3170 to 3791 °C; 04 = 3792 to 4829 °C; 05 = 4830 to 5949 °C.

There was some overlap in the fields of *s*_2_ and *s*_3_ [where *s*_3_ consisted of fields within the 90th percentile for yield among sprayed fields (*s*_3_*c*_1_); and the 90th percentile for yield among the unsprayed fields (*s*_3_*c*_2_) in the dataset], at least where high-yielding fields were concerned. All fields in *s*_3_ had predicted yields that were above the global average (Fig. 5). Yield distributions of the two cohorts within *s*_3_ were similar, with the cohorts having near-identical mean yields. Foliar fungicides contributed to between 0.1 t/ha and 0.35 t/ha to the yield increase above the global average, while the penalty (if there was one) for not using foliar fungicides was mainly confined to less than 0.05 t/ha, indicating that among the fields of *s*_3_*c*_2_ spraying was unnecessary (otherwise the penalty would have been larger). Overlaying the estimated φ values for fungicide use with MG, sowing date and growing degree days showed that these high-yielding fields were mainly in MG II and III, that the fields tended to be planted early, and were restricted to GDD groups 03 and 04 (Fig. 5), the latter factor being highly aligned with latitude. A formal comparison of the Shapley φ values across cohorts was not attempted because they potentially differed in their underlying variables despite similar yield distributions within the lowest- or highest-yield cohorts.

Intuitively, one may have expected the yield increase due to foliar fungicides to be about the same magnitude (about 0.1 t/ha) as the yield penalty associated with not using fungicides. The larger yield gain versus the penalty may be due to synergistic interactions of foliar fungicides with other management factors. For example, foliar insecticides are likely to be applied along with foliar fungicides; conversely, fields that were not sprayed with foliar fungicides were unlikely to be sprayed with insecticides as well. Therefore, in subset 4 (*s*_4_), we examined the Shapley φ values associated with fungicide use among all 210 fields in the data matrix which had been sprayed with foliar fungicides but not with foliar insecticides (*s*_4_*c*_1_), and compared them to the Shapley φ values for foliar fungicide use among another cohort of 210 fields (*s*_4_*c*_2_) which had been sprayed with both foliar fungicides and insecticides, where the fields of *s*_4_*c*_2_ were sampled to match the range of reported yields in *s*_4_*c*_1_. There was no discernable separation of the Shapley φ values between cohorts *s*_4_*c*_1_ and *s*_4_*c*_2_ (Supplementary Fig. S8 online), and the φ values were consistent with what had been observed with the other subsets of fields.

**Figure 6:**
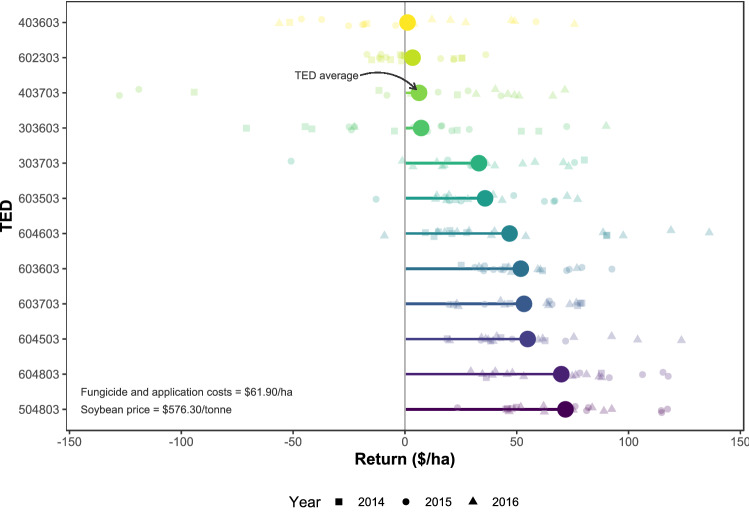
Partial economic analysis on the 20 highest-yielding, foliar fungicide-treated fields in the 12 technology extrapolation domains (TEDs) with the most fields. Return is the value of the yield increase attributed to foliar fungicides minus the cost of chemical and application. Soybean price fixed at US$576.30 per tonne (as of Jan 31 2021). Chemical and application costs fixed at US$61.90/ha. Individual fields are represented by the smaller symbols. The larger symbols are the mean returns for each TED.

A partial economic analysis estimated the net realized profit associated with foliar fungicide use on the respective cohorts within subsets of fields. The profitability of foliar fungicides in the fields of *s*_1_*c*_1_ (20 highest-yielding sprayed fields within the 12 TEDs with the most fields in the dataset) is shown in Fig. 6. It should be noted that the soybean price on which Fig. 6 is predicated reflects the high prices being experienced currently (as of Spring 2021), which are at their highest levels in at least the last five years. Assuming a price of US$576.30/t, fungicides were overwhelmingly profitable in all but four TEDs (403603, 602303, 403703, 303603) in which the average return (with respect to fungicide use) was less than US$7.50/ha. For these four TEDs, confidence intervals for the mean financial return per ha after accounting for fungicide costs indicated returns could be negative (loss), zero, or up to US$26.50/ha, depending on the individual field (Supplementary Figure S9 online). Considering these were the highest-yielding fields within TEDs, there was the risk of losing money on fungicide sprays in these four TEDs. Obviously, environment mattered (compare with Supplementary Figures S5 and S6 online), and with the four TEDs listed above the most noticeable feature was their higher-latitude locations relative to fields in other TEDs. Among other things, higher latitude is associated with cooler weather and shorter accumulation of GDD. Underlying yield potential factors (early sowing, PAWR, GDD, AI) contributed to higher predicted yield. Higher-yielding environments were more likely to also realize a larger contribution of foliar fungicides to yield above the global average (Supplementary Figure S6 online), thereby leading to the profitability of spraying.

The financial return on spraying the fields in *s*_2_*c*_2_ (100 lowest-yielding fungicide-sprayed fields) was negative, except in a few individual cases (Supplementary Figure S10 online). The mean net return due to foliar fungicides for *s*_2_*c*_1_ (100 highest-yielding fungicide-sprayed fields) was US$74.63/ha (95% CI US$69.02 to US$80.66 per ha), whereas for *s*_2_*c*_2_ the return was -US$26.24/ha (95% CI -US$33.63 to -US$19.71 per ha).

Considering the two cohorts of *s*_3_ (unsprayed and sprayed fields in the 90th percentile for yield), there was a small financial penalty to not using foliar fungicides in high-yield environments. Not spraying high-yield fields (*s*_3_*c*_2_) was associated with a mean loss of -US$10.17/ha (95% CI -US$12.50 to -US$7.83 per ha). Yet, spraying high-yield fields (*s*_3_*c*_1_) was associated with a mean gain of US$65.60 (95% CI US$61.28 to US$70.38 per ha). The trends were consistent when other soybean price points were assumed for any of the subsets examined (Supplementary Figures S11, S12, S13 online).

The soybean price required to at least break even on a (fixed) fungicide investment cost of US$61.90/ha was a nonlinear function of φ. At a realized Shapley φ value of 0.1 t/ha in response to foliar fungicides, soybean price would have to be at least US$619.00/t to recover the costs of fungicides and their application, dropping to US$309.50/t, US$206.33/t, and US$154.75/t for Shapley φ value of 0.2 t/ha, 0.3 t/ha and 0.4 t/ha, respectively.

The percentage of U.S. soybean hectarage treated with foliar fungicides rose from 1 to 11% between 2004 and 2015^42^, which is a yearly increase of 0.91%. Assuming the average gain of 0.221 t/ha due to foliar fungicides among sprayed fields in the 90th percentile for yield (*s*_2_*c*_1_), we estimated a yield gain of 2 kg ha^−1^ year^−1^ attributed to the adoption of foliar fungicide (221 × 0.91/100). This translated to 6% of the estimated annual yield gain in U.S. soybean (33 kg ha^−1^ year^−1^) attributable to foliar fungicide use in high-yield environments.

As foliar disease data were not available, we can only say that a decision was made to use foliar fungicides in about one-third of the fields, but cannot say why growers chose to spray, which could be any one of (or a combination) of cost effectiveness, perceived benefit (disease control or plant health effects) or forecast disease risk. Whatever the reason, the estimated yield gains (above the global average) attributed to foliar fungicides made spraying profitable under several soybean price scenarios, but the yield potential environment is an important consideration as highlighted by a loss on fungicide investment in some TEDs. Our finding that fungicide profitability was not universal may account for some of the discrepancies among field trials mentioned in the Introduction.

We do emphasize that foliar fungicides should not be applied indiscriminately, divorced from disease scouting or forecasting, integrated pest management and environmental principles. The price to be paid in terms of environmental damage^43^ and loss of product efficacy due to the evolution of fungicide resistance within foliar pathogen populations^44,45^ should be weighed against the yield penalty associated with not using foliar fungicides in high-yield environments. For the unsprayed fields in the 90th percentile for yield (*s*_3_*c*_2_), the average penalty associated with not spraying was 17.7 kg/ha, which works out to be US$10.62/ha at a high price of US$600/t.

## Conclusions

Most previous studies have shown little economic benefit associated with foliar fungicide application in soybean. However, our analysis, based on thousands of field observations, suggests that, except for a few production environments located in the northern fringe of the U.S. north-central region, there was an economic benefit to using foliar fungicides in soybean production when prices are near or above average. Nevertheless, foliar fungicides should always be used judiciously in an integrated program that weighs their economic benefits against their environmental consequences.

## Methods

### Soybean management database

The data matrix consisted of grower-supplied agronomic practices and average yield (adjusted to 13% moisture content) for 2738 non-irrigated soybean fields in the years 2014 to 2016 across 11 states in the U.S. north-central region: Illinois, Indiana, Iowa, Kansas, Michigan, Minnesota, Nebraska, North Dakota, Ohio, South Dakota, Wisconsin (Supplementary Fig. S1 online). The study’s data were parsed from questionnaire responses returned by soybean growers^13,36^ which, despite being survey-based, are reliable^46^. The grower-supplied data were augmented with variables representing technology extrapolation domains (TEDs) which define regions with similar climate and soils; as well with soil properties data^41^. This data structure was a fusion from different sources^47^ linked by GPS coordinates. The data used in the current study were a subset of the larger database^13,36^, and contained 20 agronomic, cultural and management practices with no missing values (the variables are listed in Supplementary Table S3 online). The data fell into 96 TEDs; the 12 TEDs with the most observed fields consisted 1688 rows (or 61.7%) of the data. Note again that the analyzed data represented rainfed (non-irrigated) soybean fields. Growers did not report on product name, chemistry, or rates of application for any of the pest control inputs they used (fungicidal, insecticidal, nematicidal, whether seed or foliar applied), and therefore the only level of detail available was whether such products were used or not.

### Basic statistical exploration

At the global level, a *t*-test was done to compare soybean yield between all fields which had been sprayed with foliar fungicides and those which had not. Separate *t*-tests comparing yields between sprayed and unsprayed fields were also done for each of the 12 TEDs with the most fields in the data matrix. A linear mixed model was fit to yield as a function of foliar fungicide use (a binary explanatory variable) with random intercept and slopes for the foliar fungicide effect within TEDs. The emphasis with these tests was on the estimation of effect size and not on *P* values, because the large number of fields in some comparisons inevitably meant very small yield differences between sprayed and unsprayed fields would have been deemed statistically significant in any case.

### Random forest modeling

The modeling workflow is shown in Supplementary Fig. S14 online. The data matrix was split (80:20) into training (2191 observations) and test (547 observations) sets. The training set was used to tune a random forest (RF) model with soybean yield as a continuous response to the 20 variables as predictors. Three RF model parameters, for the minimum number of observations in a terminal node (*min.node.size*), fraction of observations that are sampled for each tree (*sample.fraction*) and the number of candidate predictors for each split (*mtry*), were tuned simultaneously using a sequential model-based optimization strategy^48^ in the R tuneRanger package (version 0.4). Sampling was done without replacement. The tuned RF model was evaluated by predicting yield on the test set, after which it was refit to the full data matrix using the R ranger package (version 0.11.2). The number of trees was fixed at 3000 for stability in permutation-based variable importance measures^48^. The fit of the finalized RF model to the full data matrix was evaluated by plotting the residuals versus the predicted yield. The RF model was then interpreted using model-agnostic approaches^39,49^.

### Global model interpretation

A permutation-based approach was used to assess feature (predictor) importance^50^. This approach is more principled than the Gini impurity score because Gini-based metrics are biased with RF models^50^. In this method, each feature’s values are permuted (shuffled) and then the loss in model performance is measured. Those features which are important will be associated with a larger drop in model performance compared to that for features that are not as important in predicting soybean yield. Performance loss was measured by the mean absolute error (mean squared error is another choice). Importance was summarized by the ratio (*FI* = *err*_*p*_/*err*_*o*_) of the model error after permuting the feature (*err*_*p*_) to the original model error (*err*_*o*_). The permutations were repeated 20 times. Feature importance was summarized visually by plotting the median of *FI*, and the 5% and 95% quantiles.

### Local model interpretation

Shapley values (φ) are an application of coalitional (cooperative) game theory to machine learning^51^. In the present context, the goal was to compute the contributions of the features based on the difference between the predicted yield for a single field and the global average, with an emphasis on the impact of foliar fungicide use in soybean fields. For any one observation, the φ values are an estimate of how much a predictor contributed to the difference between an individual field’s predicted yield and the predicted yield averaged across all fields in the data matrix. In other words, say the predicted yield for field *i* is *x*_*i*_ above the global average. Shapley values estimate the average marginal contribution of each feature to *x*_*i*_, with the understanding that not all features (for that field) may have contributed equally, if at all, and that some may have contributed negatively. Estimating Shapley values exactly is a computationally expensive process^49^, and for this study they were approximated via Monte Carlo sampling (1000 iterations for each field) as implemented in the *Shapley* function of the R iml package (version 0.9.0).

We studied the Shapley values within different subsets (*s*) of fields in the data matrix, consisting of different cohorts (*c*) described as follows (see Supplementary Table S1 online). In subset 1 (*s*_1_), cohorts were selected from each of the 12 TEDs with the most fields in the data matrix, where within each of those TEDs the 1st cohort consisted of the 20 highest-yielding fields among those sprayed with foliar fungicides (*s*_1_*c*_1_) and the 2nd cohort consisted of the 20 highest-yielding fields among those which were not sprayed (*s*_1_*c*_2_). The four cohorts of subset 2 were the 100 highest-yielding fungicide-treated (sprayed) fields (*s*_2_*c*_1_), the 100 lowest-yielding sprayed fields (*s*_2_*c*_2_), the 100 highest-yielding unsprayed fields (*s*_2_*c*_3_), and the 100 lowest-yielding unsprayed fields (*s*_2_*c*_4_), among all fields. There were two cohorts in subset 3, chosen from all fields in the data matrix: the 90th percentile for yield among sprayed fields (*s*_3_*c*_1_); and the 90th percentile for yield among the unsprayed fields (*s*_3_*c*_2_). A final subset (*s*_4_) consisted of two cohorts, the first being the 210 fields which had been sprayed with foliar fungicides but not with foliar insecticides (*s*_4_*c*_1_). The second cohort of *s*_4_ (*s*_4_*c*_2_) was made up of a random sample of 210 of the 623 fields which had been sprayed with both foliar fungicides and foliar insecticides, with yields restricted to be within the range of yields in *s*_4_*c*_1_.

For each of the defined subsets, the φ values associated with foliar fungicide use were plotted against the difference between the predicted yield for the field and the global yield average. The distributions of the φ values within each cohort was also plotted.

The φ values associated with foliar fungicide use were interpreted as follows. If foliar fungicide applications had no effect, then the φ value for that feature would be zero for the field. If the predicted yield for a sprayed field was greater than the global average yield, then a positive fungicide φ value was an estimate of how much of the yield increase (above the global average) was due to fungicide application. If, however, a sprayed field’s yield was below the global average then a positive fungicide φ value estimated how much the spray contributed to raising the yield in a situation in which other features contributed more heavily to a yield reduction (to below the global average). That is, the fungicide was not able to counterbalance the negative effects that other features had on yield. For any sprayed field, a negative fungicide φ value would indicate a yield reduction (loss) due to spraying, perhaps due to very high disease pressure or wheel damage^6^. Finally, for unsprayed fields a positive φ value for the fungicide feature would counterintuitively indicate that yield benefitted from *not* spraying, whereas a negative φ value for the fungicide feature would estimate how much yield was penalized by not applying a foliar fungicide.

The complete code for the analysis, including other global (ALE, ICE) and local (LIME) interpretation methods (shown in Supplementary Figure S14) is provided at https://github.com/PSUPlantEpidemiology/ML_Soybean_ScientificReports/tree/v1.0).

### Economic return to foliar fungicides

The Shapley φ values associated with foliar fungicide use were used in a partial economic analysis to estimate the net profit (loss) realized by applying foliar fungicides to the soybean crop. Soybean price (*price*) was fixed at the price as of Jan 31, 2021 (US$576.30/t). The combined cost of fungicide plus its application (*chem.cost*) was also held fixed, at US$61.90/ha^19^. For unsprayed fields, *chem.cost* = 0. As the φ values are on the same scale as yield (i.e., t/ha), the net value (*net.val*) on the yield increase (loss) due to foliar fungicides was *net.val* (US$/ha) = *price* × φ. Then the net profit (*net.profit*) associated with spraying the soybean crop with foliar fungicides was *net.profit* (US$/ha) = *net.val*–*chem.cost*. Bias corrected and accelerated (BC_a_) bootstrap confidence intervals^52^ were calculated for mean net profit estimates.

Setting *net.profit* = 0 and solving for *price* gave the minimum soybean price required to break even on the costs of foliar fungicide applications given a realized φ value. That is, the break-even *price* (*price*_0_) was given by *price*_0_ = *chem.cost*/φ. This latter equation showed that *price*_0_ was a nonlinear decreasing function of φ, conditional on *chem.cost* being fixed. *price*_0_ was estimated for different φ values represented by the cohorts.

## Supplementary Information

Supplementary Information.
