# Supplementary material for: A machine learning interpretation of the contribution of foliar fungicides to soybean yield in the north‐central United States
Source: Sci Rep. 2021 Sep 21;11:18769. doi: 10.1038/s41598-021-98230-2 (PMC8455673; doi:10.1038/s41598-021-98230-2)
Supplement: Supplementary file 1 — Supplementary Information. [file 41598_2021_98230_MOESM1_ESM.docx]

Table S1. Definitions of subsets and cohorts of fields.

| **Acronym** | **TED subset** | **Fungicide use**^a^ | **Yield** | **Subset (*s*)** | **Cohort (*c*)** |
| --- | --- | --- | --- | --- | --- |
| HYF | Top 12^b^ | Yes | 20 highest-yielding fields per TED | 1 | 1 |
| HYNF | Top 12 | No | 20 highest-yielding fields per TED | 1 | 2 |
| AllHYF | All TEDs | Yes | 100 highest-yielding fields | 2 | 1 |
| AllLYF | All TEDs | Yes | 100 lowest-yielding fields | 2 | 2 |
| AllHYNF | All TEDs | No | 100 highest-yielding fields | 2 | 3 |
| AllLYNF | All TEDs | No | 100 lowest-yielding fields | 2 | 4 |
| HQF | All TEDs | Yes | 90^th^ percentile for yield | 3 | 1 |
| HQNF | All TEDs | No | 90^th^ percentile for yield | 3 | 2 |
| YFNI^c^ | All TEDs | Yes | . | 4 | 1 |
| YFYI^d^ | All TEDs | Yes | . | 4 | 2 |

^a^ No = fields were not sprayed with foliar fungicides. Yes = fields were sprayed with foliar fungicides at least once.

^b^ The 12 technology extrapolation domains (TEDs) with the most number of fields in the analyzed dataset. See Table S2.

^c^ The subset of fields sprayed with foliar fungicides but not with foliar insecticides.

^d^ A subset of fields sprayed with foliar fungicides and with foliar insecticides.

Table S2. The 12 technology extrapolation domains (TEDs) with the highest number of fields in the analyzed dataset. The total number of fields per TED is subset by the number that were sprayed with foliar fungicides or not sprayed.

|  | **Number of fields** | | |  | **Mean yield (t/ha)** | |
| --- | --- | --- | --- | --- | --- | --- |
| **TED**^a^ | **Sprayed** | **Unsprayed** | **Total** |  | **Sprayed** | **Unsprayed** |
| 603703 | 106 | 126 | 232 |  | 4.06 | 3.95 |
| 603603 | 79 | 142 | 221 |  | 4.19 | 3.80 |
| 604603 | 71 | 141 | 212 |  | 4.25 | 4.32 |
| 602303 | 20 | 122 | 142 |  | 2.81 | 2.62 |
| 504803 | 47 | 92 | 139 |  | 4.59 | 4.09 |
| 604803 | 61 | 66 | 127 |  | 4.54 | 4.11 |
| 604503 | 27 | 85 | 112 |  | 4.54 | 3.73 |
| 303603 | 20 | 87 | 107 |  | 3.98 | 3.73 |
| 303703 | 22 | 84 | 106 |  | 4.32 | 3.55 |
| 603503 | 46 | 59 | 105 |  | 4.26 | 4.07 |
| 403703 | 21 | 73 | 94 |  | 3.88 | 3.68 |
| 403603 | 17 | 74 | 91 |  | 3.79 | 3.58 |

^a^ There were 96 TEDs in the analyzed dataset. All other TEDs had less than 75 fields.

Table S3. Grower-reported management and soils variables used as predictors of soybean yield.

| **Feature** | **Description** | **Units, mean, range or categories** |
| --- | --- | --- |
| doy | Sowing date as the number of days from Jan 01 | 100 - 196 |
| latitude | Field’s latitude coordinate | 37.11N - 48.97N |
| pH.0.30.cm | pH of the topsoil (to a depth of 30cm) | 5.03 - 8.23; mean = 6.64 |
| OM.0.30.cm | Percent organic matter in the topsoil (0 to 30 cm depth). | 0.5% - 31.6%; mean = 3.4% |
| seed.rate | Seeding rate (no. seeds sown per ha) | 222.4K - 556K; mean = 384.4K |
| GDD | Annualized growing degree days for the technology extrapolation domain (TED) in which the field was located; five categories | 01 = 0 - 2,670℃  02 = 2,671 - 3,169℃  03 = 3,170 - 3,791℃  04 = 3,792 - 4,829℃  05 = 4,830 - 5,949℃ |
| foliar.fungicide | Binary variable for whether the soybean field was treated with foliar fungicides or not | N = unsprayed  Y = fungicide-treated |
| AI | TED-construct annual aridity index (AI; unitless) for the TED in which the field was located; eight categories | 1 = 2696 - 3893  2 = 3894 - 4791  3 = 4792 - 5689  4 = 5690 - 6588  5 = 6589 - 7785  6 = 7786 - 8685  7 = 8686 - 10181  8 = 10182 - 12876 |
| MG | Soybean maturity group; five categories | 0, I, II, III, IV |
| foliar.insecticide | Binary variable for whether the soybean field was treated with foliar insecticides or not | N = unsprayed  Y = insecticide-treated |
| texture.0.30.cm | Texture of the topsoil (0 to 30 cm depth) according to the USDA soil texture classes; five categories | Silt loam  Silty clay loam  Clay loam  Sandy loam  Loamy sand |
| TWI | Topographic wetness index (unitless) | 6.899 - 12.378; mean = 9.898 |
| PAWR | Plant available water holding capacity in the rooting zone; seven categories | 1 = 0 - 50 mm  2 = 50 - 100 mm  3 = 100 - 150 mm  4 = 150 - 200 mm  5 = 200 - 250 mm  6 = 250 - 300 mm  7 = 300+ mm |
| herbicide | Categorical variable for the type of herbicide treatment used on the field; four categories | pre = pre-emergent only  post = post-emergent only  both = pre- and post-emergent products used  none = no herbicides used |
| row.space | Row spacing at planting; five categories | row7 = 7 - 8 inch (17.78 - 20.32 cm)  row10 = 10 inch (25.4 cm)  row15 = 15 inch (38.1 cm)  row22 = 22 inch (55.88 cm)  row30 = 30 inch (76.2 cm) |
| seed.trt1 | Binary variable for whether a seed treatment (any of fungicide, insecticide, nematicide, growth regulator or inoculant) was used | N = no seed treatment  Y = seed was treated |
| starter.fert | Binary variable for whether a starter fertilizer was used | N = no starter fertilizer was used  Y = a starter fertilizer was used |
| lime | Binary variable for whether lime was applied to the field in the planting year or up to three years prior | N = no lime was applied  Y = field was limed |
| manure | Binary variable for whether manure was applied to the field in the planting year or up to three years prior | N = no manure was applied  Y = manure applied to the field |
| iron.def | Binary variable for whether iron deficiency chlorosis was noticeable in the soybean crop | N = no symptoms of iron deficiency  Y = symptoms of iron deficiency present |


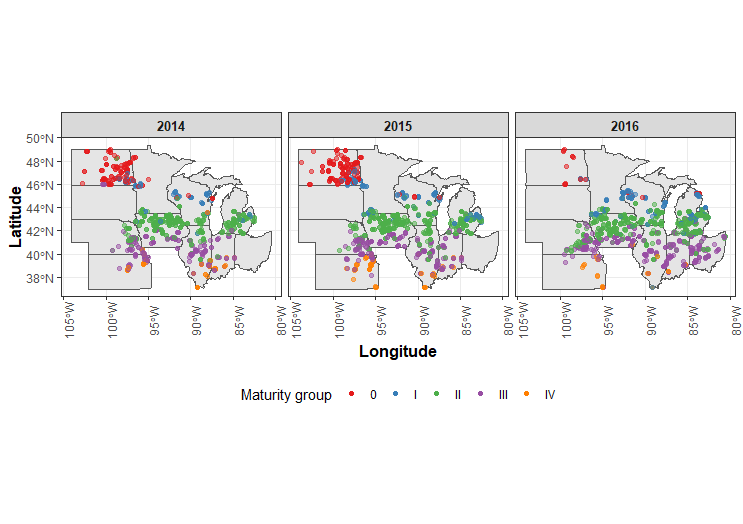


Supplementary Figure S1. Locations of soybean fields for which surveyed growers supplied self-reported data on their management practices and yields, 2014 to 2016. Field locations are colored by soybean maturity group.


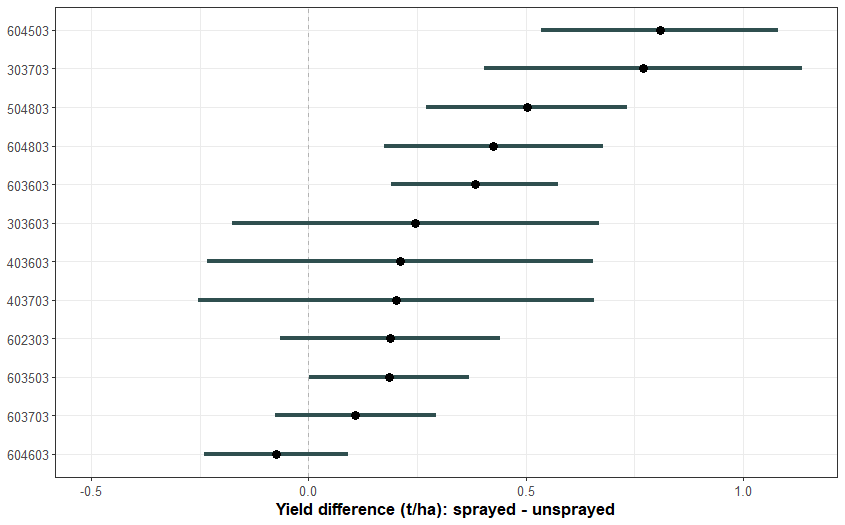


Supplementary Figure S2. *t*-test estimates of the yield difference in yield between soybean fields sprayed with foliar fungicides and those which had not been sprayed, for 12 technology extrapolation domains (TEDs). Points represent the mean estimate. Bars are the 95% confidence interval for the mean.


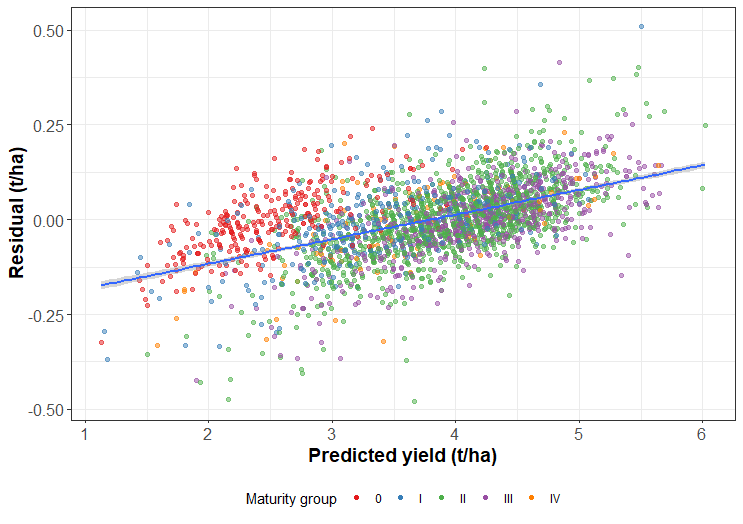
 Supplementary Figure S3. Residuals versus predicted yield for a random forest model fit to grower-reported soybean yield as a function of management variables. Points are colored by soybean maturity group. The line is a linear regression of the residuals on predicted yield.


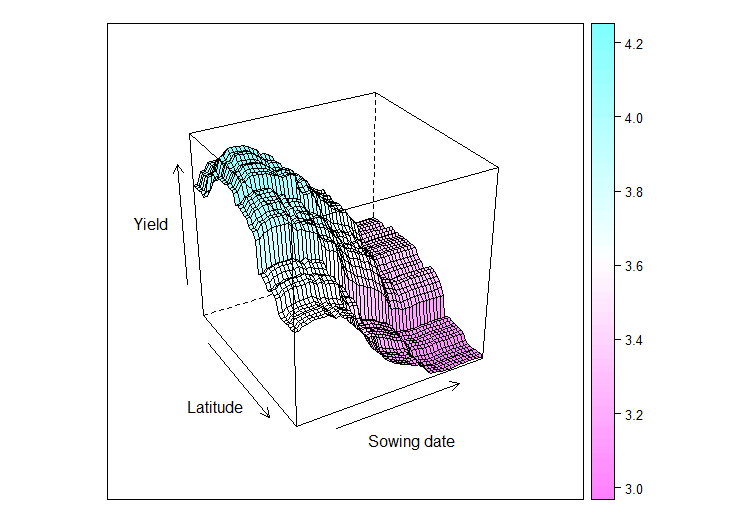


Supplementary Figure S4. Two-way partial dependence plot of the global model effects of latitude and sowing date (day of year) on soybean yield (t/ha).


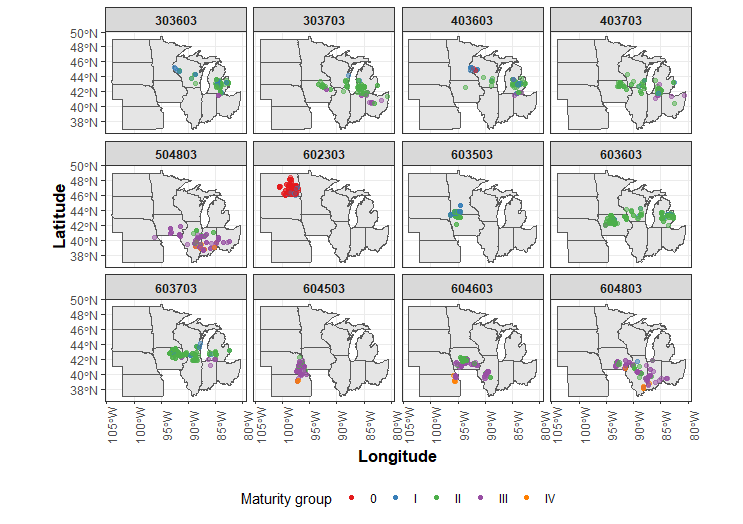


Supplementary Figure S5. Locations of soybean fields stratified by technology extrapolation domains (TEDs), for the 12 TEDs with the most fields in the dataset. Locations are colored by soybean maturity group.


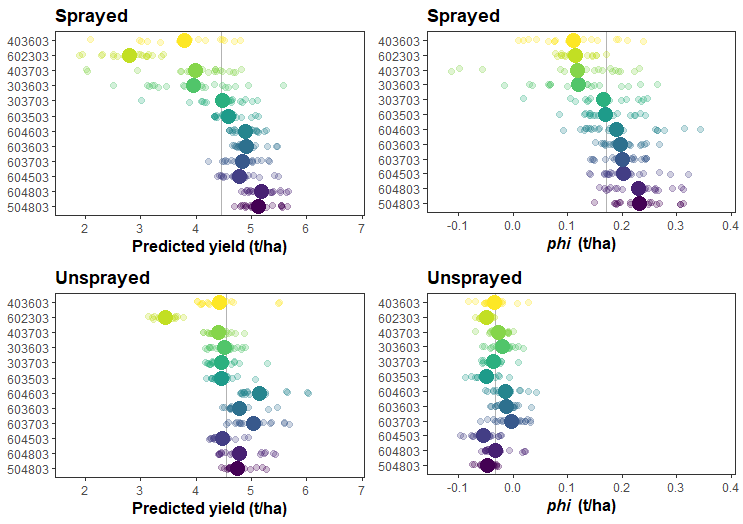


Supplementary Figure S6. Summaries of the predicted yields and Shapley φ (*phi*) values for the two cohorts of fields shown in Fig. 3. The cohorts are the 20 highest-yielding fields among those sprayed with foliar fungicides and the 20 highest-yielding fields among those which were unsprayed, in each of the 12 technology extrapolation domains (TEDs) with the most fields out of all TEDs represented in the data set (subset 1 cohorts 1 & 2; Supplementary Table S1). The gray vertical line is the overall mean of either predicted yield or the *phi* values across all fields shown in the panel.


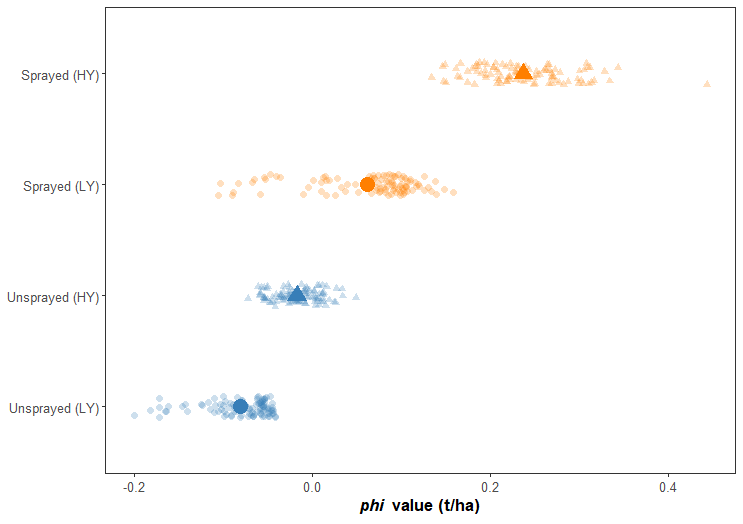


Supplementary Figure S7. Summary of the Shapley *phi* values attributed to foliar fungicide use for four cohorts of soybean fields shown in Fig. 4. The cohorts are Sprayed (HY): the 100 highest-yielding fungicide-treated fields; Sprayed (LY): the 100 lowest-yielding fungicide-treated fields; Unsprayed (HY): the 100 highest-yielding unsprayed fields; and Unsprayed (LY): the 100 lowest-yielding unsprayed fields (subset 2 cohorts 1 to 4; Supplementary Table S1). The jittered points represent the individual fields. The larger symbols are the means for the respective cohorts.


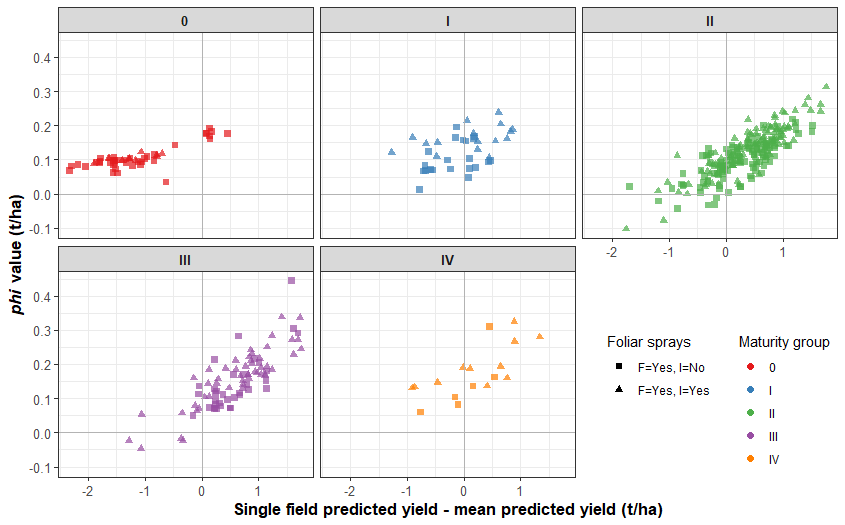


Supplementary Figure S8. Shapley *phi* values attributed to foliar fungicide use for the two cohorts of fields of subset 4 (Table S1). The first cohort (square symbols) are the 210 fields in the data matrix which had been sprayed with foliar fungicides (F) but not with foliar insecticides (I). The second cohort (triangle symbols) was a random sample of 210 fields from those which had been sprayed with both foliar fungicides and insecticides, restricted to the range of reported yields in the first cohort.


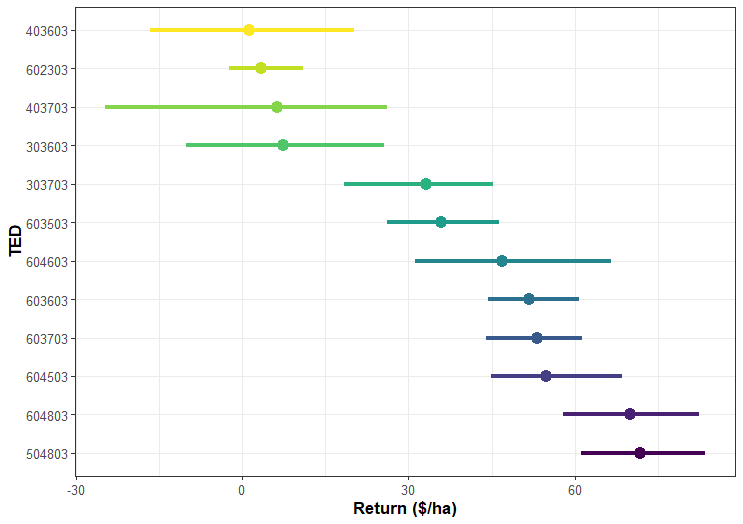


Supplementary Figure S9. Mean financial return and 95% confidence interval on foliar fungicide use in the highest-yielding soybean fields, by technology extrapolation domain (TED). Returns assume a price of US$576.30 per tonne, and a combined fungicide product and application cost of US$61.90 per ha.


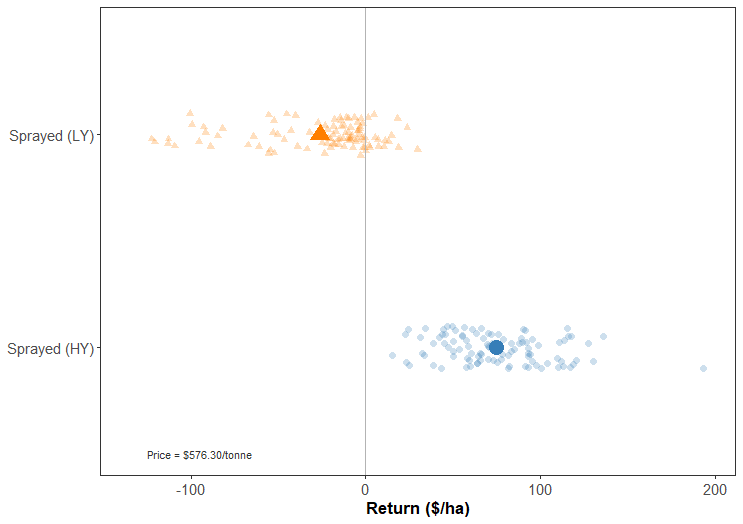


Supplementary Figure S10. Financial return attributed to foliar fungicide use on the 100 highest-yielding (HY) and the 100 lowest-yielding (LY) fields, all fields having been sprayed with fungicides (subset 2 cohorts 1 & 2; Supplementary Table S1). Returns assume a price of US$576.30 per tonne, and a combined fungicide product and application cost of US$61.90 per ha. The smaller symbols represent individual fields. The larger symbols are the means for the respective cohorts.


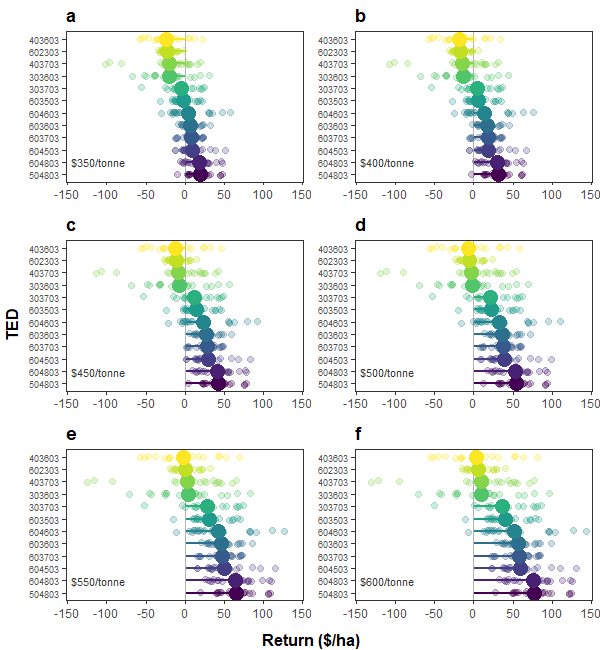


Supplementary Figure S11. Partial economic analysis on the 20 highest-yielding, foliar fungicide-sprayed fields in the 12 technology extrapolation domains (TEDs) with the most fields. Return is the value of the yield increase attributed to foliar fungicides minus the cost of chemical and application. Returns are shown for six different soybean price points. Chemical and application costs fixed at US$61.90/ha.


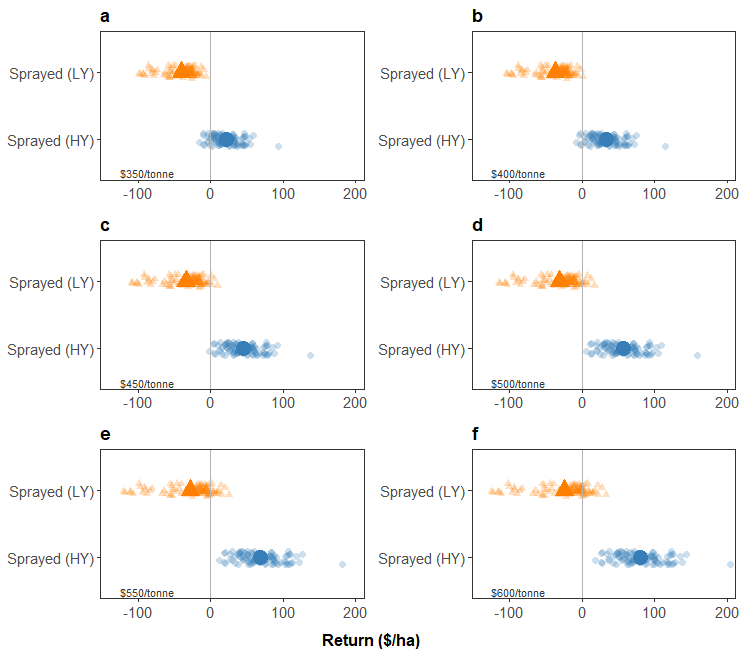


Supplementary Figure S12. Financial return attributed to foliar fungicide use on the 100 highest-yielding (HY) and the 100 lowest-yielding (LY) fields, all fields having been sprayed with fungicides (subset 2 cohorts 1 & 2; Supplementary Table S1). Returns assume a combined fungicide product and application cost of US$61.90 per ha. Panels represent different soybean price points ranging from US$350 per tonne to US$600 per tonne. The smaller symbols represent individual fields. The larger symbols are the means for the respective cohorts.


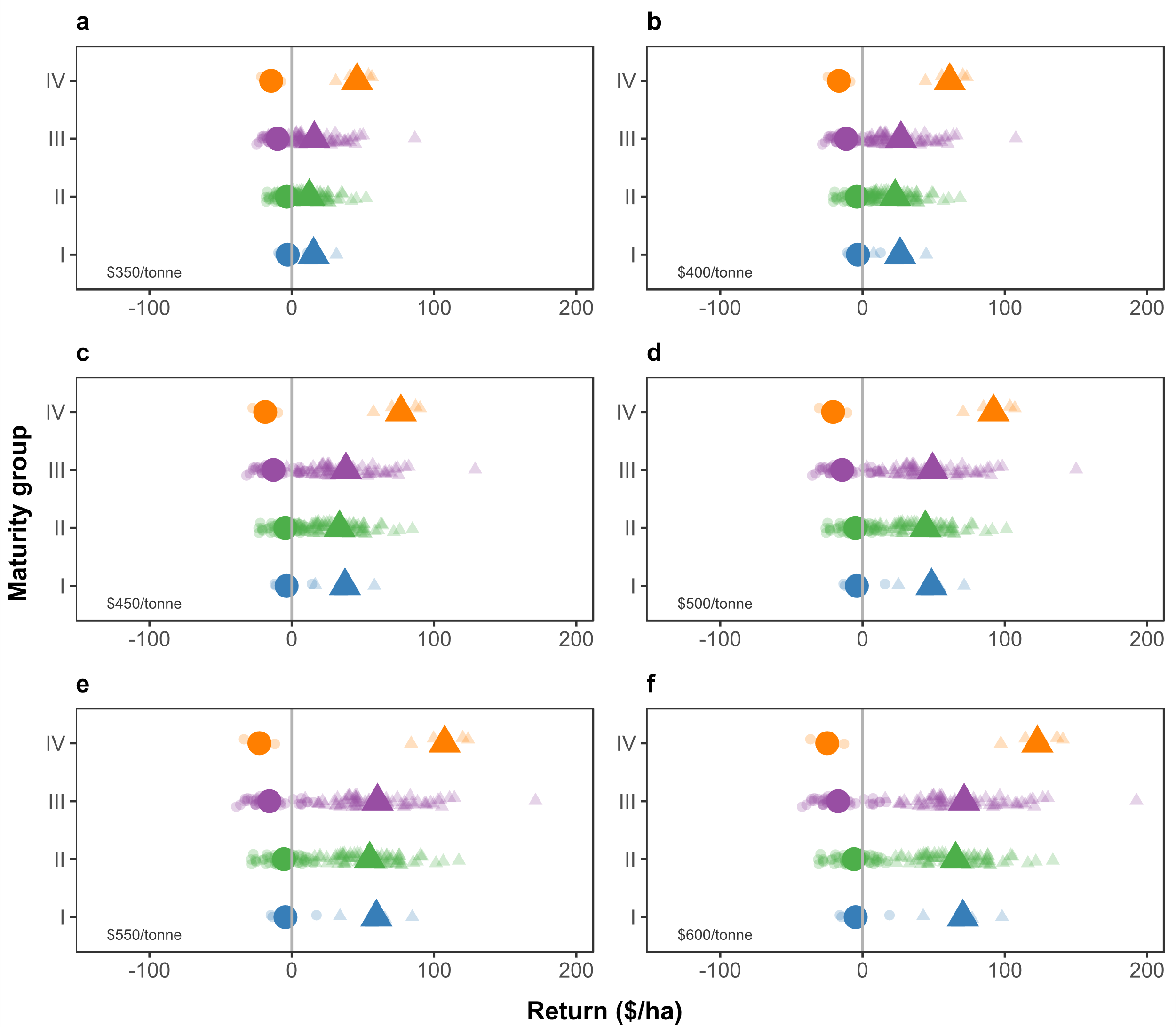


Supplementary Figure S13. Financial return (loss) attributed to foliar fungicide use (non-use) on two cohorts (subset 3; Supplementary Table S1) of high-yielding soybean fields: the 90^th^ percentile for yield among sprayed fields (triangle symbols), and the 90^th^ percentile for yield among unsprayed fields (circle symbols). Smaller symbols represent individual fields. Larger symbols are the respective means. Returns assume a combined fungicide product and application cost of US$61.90 per ha, which is zero in the case of unsprayed fields. Panels represent different soybean price points ranging from US$350 per tonne to US$600 per tonne.


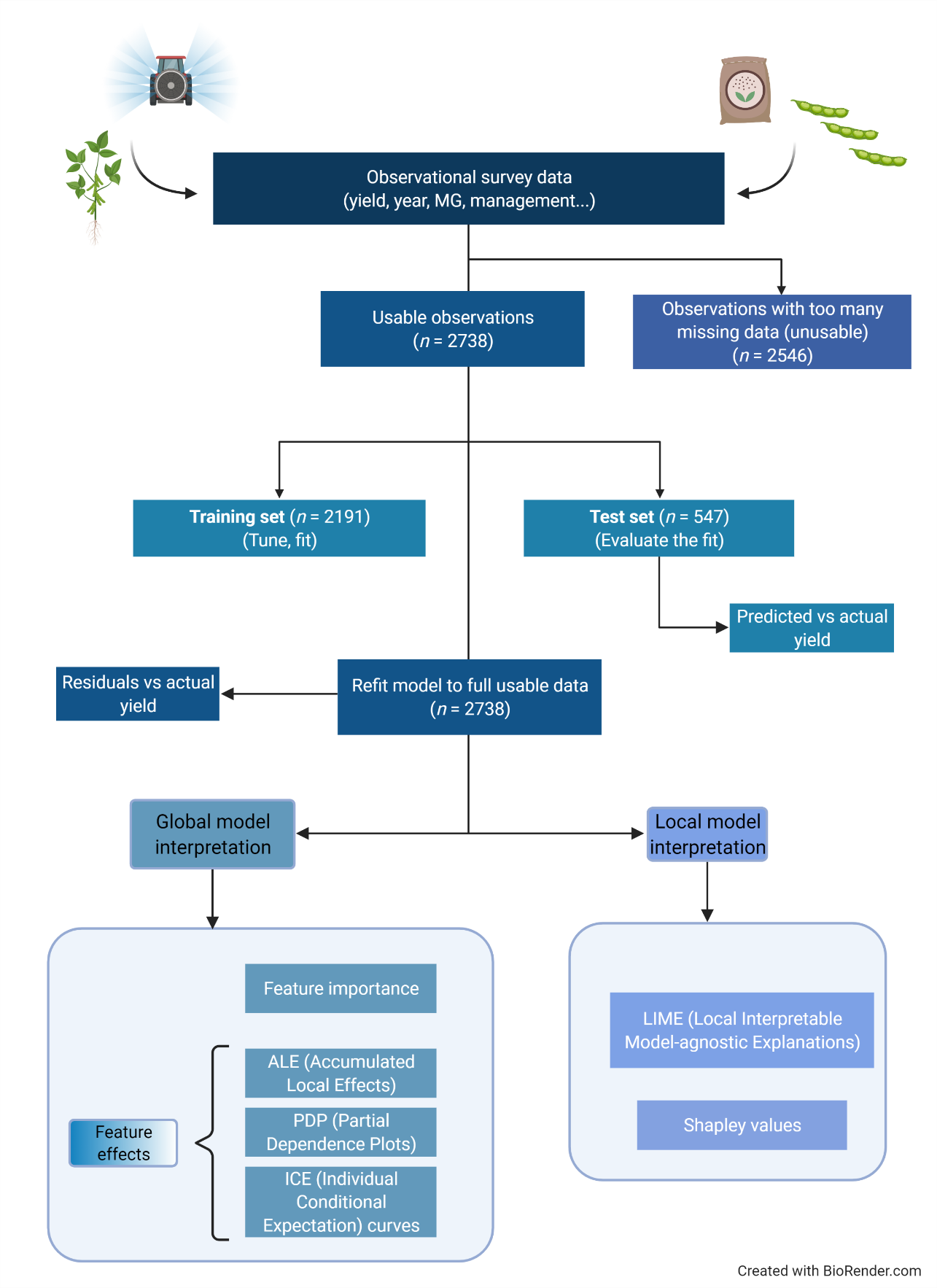


Supplementary Figure S14. Schematic of the analytical steps. ALE, ICE and LIME interpretations are not shown in the paper but are provided at https://github.com/PSUPlantEpidemiology/ML_Soybean_ScientificReports/tree/v1.0.
